# An Internet-based intervention for eating disorders consisting of automated computer-tailored feedback with or without supplemented frequent or infrequent support from a coach: study protocol for a randomized controlled trial

**DOI:** 10.1186/1745-6215-14-340

**Published:** 2013-10-17

**Authors:** Jiska J Aardoom, Alexandra E Dingemans, Philip Spinhoven, Leona Hakkaart-van Roijen, Eric F Van Furth

**Affiliations:** 1 Center for Eating Disorders Ursula, Postbox 422, 2260 AK, Leidschendam, The Netherlands; 2 Institute of Psychology, Leiden University, Wassenaarseweg 52, 2333 AK, Leiden, the Netherlands; 3 Department of Psychiatry, Leiden University Medical Center, Postbox 9600, 2300 RC, Leiden, the Netherlands; 4 Institute of Health Policy and Management (iBMG) and institute for Medical Technology Assessment (iMTA), Erasmus University Rotterdam, Postbus 1738, 3000 DR, Rotterdam, the Netherlands

**Keywords:** Computer-tailored, Eating disorders, Internet, Intervention, Predictors, Prevention, Self-help, Therapist support, Treatment

## Abstract

**Background:**

Several Internet-based interventions for eating disorders have shown their effectiveness. Still, there is a need to refine such interventions given that most existing programs seem to be limited by their static ‘one-size-fits-all’ approach. ‘Featback’, an Internet-based intervention for symptoms of eating disorders provides a more individualized approach. It consists of several components (psychoeducation, a fully automated monitoring and feedback system, and support from a coach), which can be matched to participants’ needs and preferences. Until now, it is unclear whether online self-help interventions for eating disorders with support are more effective than those without. The aims of the current study are i) to examine the relative effectiveness of (the different components of) Featback; ii) to examine predictors, moderators and mediators of intervention responses; iii) to report on practical experiences with Featback; and iv) to examine the cost-effectiveness of Featback.

**Methods/design:**

Individuals aged 16 years or older, with mild to severe eating disorder symptoms will be randomized to one of the four study conditions. In condition one, participants receive the basic version of Featback, consisting of psychoeducation and a fully automated monitoring and feedback system. In conditions two and three, participants receive the basic version of Featback supplemented with the possibility of infrequent (weekly) or frequent (three times a week) e-mail, chat, or Skype support from a coach, respectively. The fourth condition is a waiting list control condition. Participants are assessed at baseline, post-intervention (8 weeks), and at 3- and 6-month follow-up (the latter except for participants in the waiting list control condition). Primary outcome measures are disordered eating behaviors and attitudes. Secondary outcome measures are (eating disorder-related) quality of life, self-stigma of seeking help, self-esteem, mastery and support, symptoms of depression and anxiety, repetitive negative thinking, motivation to change, user satisfaction, compliance, and help-seeking attitudes and behaviors.

**Discussion:**

This study aims to provide more insight into the (cost-) effectiveness of Internet-based interventions for eating disorders, particularly those with and without professional support, as well as different levels of support.

**Trial registration:**

NTR3646

## Background

Despite the disabling nature of mental disorders, many individuals with mental health problems do not receive treatment [1]. Suggested reasons for this treatment gap include stigma, embarrassment, lack of recognition of symptoms, and preference for self-reliance [2,3]. New technologies can possibly bridge the gap between the need and the actual received treatment, by providing ways to reach individuals who are otherwise hard to reach. For example, the anonymity of Internet-based interventions can decrease barriers that exist in more intensive face-to-face treatment. Internet-based interventions also offer other advantages over traditional face-to-face interventions, such as cost-effectiveness and widespread dissemination. Accessibility and convenience can be enhanced as Internet-based interventions are available 24 hours a day and can be accessed at any place. It is not surprising that the field of e-mental health is rapidly growing: more and more Internet-based interventions for mental disorders have been developed and investigated over the past years. Numerous programs have proven to be effective, particularly in preventing and reducing symptoms of depression and anxiety [4-6].

In the field of eating disorders (ED), a recent review demonstrated the superiority of Internet-based therapy over waiting lists for the reduction of ED psychopathology, the frequency of binge eating and purging, and also for the improvement of (ED-related) quality of life [7]. Regarding the preventive intervention of ED, numerous studies have evaluated an Internet-based intervention called ‘Student Bodies’ [8-15], consisting of psychoeducation, a web-based body image journal (allowing participants to monitor events that trigger body image dissatisfaction), and an online asynchronous discussion group. A meta-analytic review [16] demonstrated ‘Student Bodies’ to be effective in reducing ED-related attitudes, such as weight and shape concerns and a negative body image.

Despite these promising findings, there is a need to refine the Internet-based interventions for ED as the existing interventions seem to be limited by their static, ‘one-size-fits-all’ approach. The Internet-based program ‘Es[s]prit’ [17] possibly constitutes a step forward. It combines prevention and (early) intervention of ED and consists of several components: psychoeducation, a fully automated symptom monitoring and feedback system, a forum, and chat sessions (either individual or in group) with a coach. The use and intensity of the components can be adapted according to the participant’s needs, a so-called ‘stepped-care approach’. The core module of the program is the monitoring and feedback system. Once a week, participants are invited to complete a monitoring assessment which appraises ED-related attitudes and behaviors. Subsequently, automatic feedback messages are generated and send to participants. The feedback messages do not only provide support by expressing interest in, and concerns about the participants’ well-being, but also contain advice on how to counteract negative developments in ED-related symptoms.

Es[s]prit was developed in Germany [17] and has been translated and adapted into several other languages. Until now, preliminary studies of Es[s]prit show promising results and suggest the intervention to be feasible and acceptable for college students [17,18], as well as for women who completed inpatient or outpatient treatment for bulimia nervosa or ED not otherwise specified [19]. Furthermore, Lindenberg et al. (personal communication) investigated the effectiveness of YoungEs[s]prit, primarily focusing on the prevention of ED in high-school students aged 13–16. The results demonstrated that within one year, the incidence rate of ED was significantly lower in the group of students who received the YoungEs[s]prit intervention as compared to a control group. There is a need to systematically investigate the Dutch translation of Es[s]prit, called ‘Featback’, focusing on both the prevention and intervention of ED. Moreover, the effectiveness of the different components of Featback (and thus the different levels of support) has yet to be established, and no cost-effectiveness analysis of the program has been conducted.

The evaluation of the necessity and importance of adding more personalized levels of support is important as it is associated with an increase in costs and may furthermore limit the availability of the intervention, given the need for a sufficient number of coaches. Nevertheless, providing personalized support seems to be beneficial: studies in the field of depression and anxiety suggest that Internet-based interventions with support are more effective than those without [4,6]. However, the degree of provided support varies considerably and the most adequate quantity of support in order to achieve positive effects of an intervention is yet unknown. Two Internet-based intervention studies, one for social phobia [20] and one for panic disorder [21], failed to identify an incremental effect of more frequent levels of support, although both studies had small sample sizes. Tate et al. [22] compared e-mail counseling, computer-automated tailored counseling, and no counseling in an Internet weight loss program. After six months, participants in the e-mail counseling group achieved significantly greater weight loss than participants in the computer-automated feedback. Given these mixed results on the necessity and importance of personalized support, the current study could help to gain more insight in the potential incremental effects of different levels of support on the effectiveness of an intervention.

This randomized controlled trial will compare four conditions: i) a basic version of Featback consisting of psychoeducation and a fully automated monitoring and feedback system; ii) same as (i), but supplemented with the possibility of infrequent, weekly e-mail, chat or Skype support from a coach; iii) same as (i), but supplemented with the possibility of frequent (three times a week) e-mail, chat or Skype support from a coach; and iv) a waiting list control condition (WLC). The WLC could be regarded as a care-as-usual condition, given that all participants are free to undergo any other intervention. The three active intervention conditions are developed in a stepped-care framework, starting with the least intensive intervention and moving up to (the possibility of) more intensive components. Participants are assessed at baseline (T0), post-intervention (T1: 8 weeks), and at 3- (T2) and 6-month follow-up (T3). Participants in the WLC condition will not be measured at T3, as they will be offered the intervention of condition two after T2.

The primary aim of the current study will be to investigate the effectiveness of (the different components of) Featback. The second aim is to investigate potential predicting, moderating, and mediating variables in order to gain insight into when or for whom this intervention works, as well as how it works. The third aim of this study is to report on the practical experiences of Featback, such as the user satisfaction and the (intensity of) use of the different components. Finally, the fourth aim of this study is to examine the cost-effectiveness of Featback.

## Methods

### Design

This study is a randomized control trial including three active intervention conditions and a waiting list control condition (for more details: see Study conditions). Ethical approval has been obtained by an independent medical ethics committee (CCMO no. NL40085.058.12).

### Sample

Inclusion criteria will be deliberately kept broad, given that we aim to reach a broad population of individuals with ED. Participants will have to: i) be sixteen years of age or older; ii) report at least mild ED symptoms (as assessed by the Short Evaluation of Eating Disorders (SEED) [23]) or show at least some risk for the development of an ED (as defined by scoring 40 or higher on the Weight Concern Scale (WCS) [24]); and iii) have access to a computer, iPhone, iPad, Smartphone or laptop with an Internet connection. Regarding the second criterion (reporting at least mild ED symptoms), individuals need to report at least one of the following symptoms: a BMI of 18.5 or less, self-induced vomiting, binge eating episodes, excessive exercise or use of laxatives for at least once a week over the past four weeks, or a body distortion showing that one’s estimated BMI is at least two points higher than one’s actual BMI.

### Procedure and randomization

Participants will be recruited through advertisements on websites (among others Proud2Bme (http://www.Proud2bme.nl)), and/or academic schools, magazines, newspapers, health care centers, and patient unions. Interested individuals can apply for participation by sending an e-mail to the researcher, who will send them an information letter and invite them to complete an online informed consent and screening questionnaire. After completion, individuals receive an e-mail with feedback on the severity of their ED symptoms, and those who meet the inclusion criteria will be invited to complete the baseline questionnaire for the study. After completion of the baseline questionnaire, participants will be randomized to one of the four study conditions and will be notified by e-mail. The randomization allocation will be conducted by an independent researcher, who will create random-number tables by means of SPSS. Randomization will take place in blocks of 40 participants. The number of participants (n = 10) in each block will be equal for the four conditions. The allocation sequence will be concealed from the main researcher involved in the enrolment and assignment of participants, thus preventing foreknowledge of the intervention assignment.

### Study conditions

#### Basic featback

Participants will receive access to the website of Featback (http://www.Featback.nl) where comprehensive information on ED can be found (e.g., psychoeducation). In addition, participants will have access to a monitoring and feedback system. On a weekly basis, participants will receive an e-mail inviting them to complete a monitoring questionnaire. This monitoring questionnaire consists of eight items assessing cognitive and behavioral correlates of four dimensions: i) body dissatisfaction; ii) excessive concern with body weight and shape; iii) unbalanced nutrition and dieting; and iv) binge eating and compensatory behaviors. Answers can be given on a 4-point Likert scale. After completion of the monitoring assessment, feedback messages are automatically generated according to a pre-defined algorithm. The feedback messages are individually tailored. That is, they are based on the functionality of reported ED-related attitudes and behaviors (functional versus dysfunctional), as well as patterns of change (improved, deteriorated or unchanged). For detailed information about the feedback algorithm, see Bauer et al. [17]. Subsequently, the generated feedback is sent to the participants using Web-Akquasi data management software [25]. In case of severe ED symptoms, action is taken (see ‘Ethical precautions and crisis management’ for more details).

#### Basic featback + infrequent support

Participants will receive the basic Featback intervention as described above, supplemented with the possibility of infrequent (weekly) e-mail, chat or Skype support from a coach.

#### Basic featback + frequent support

Participants will receive the basic Featback intervention as described above, supplemented with the possibility of frequent (three times a week) e-mail, chat or Skype support from a coach.

#### Waiting list control condition (WLC)

Participants in this condition will be assigned to a waiting list, for the purpose of providing a comparison group for the active intervention conditions. Participants will be offered the intervention of condition two after a waiting period of five months (T2).

### Support from a coach

Participants will be able to schedule support sessions through different mediums: e-mail, chat or Skype. Coaches will be instructed to e-mail participants in case participants do not schedule any appointments or in case participants do not show up at scheduled support sessions, and to repeat this process twice per non-response. Chat and Skype sessions will imply 20 minutes with a coach. An e-mail session will contain one reply by e-mail from the coach to the participant (who will be instructed to e-mail his/her coach beforehand). The methodology of chat sessions is based on a 5-phase model, containing i) a warm welcome, ii) clarifying of the question, iii) determining the goal of the conversation, iv) concrete elaboration of the goal of the conversation, and v) closing the circle. More detailed information on this model can be found in the handbook written by Schalken et al. [26]. E-mail support is based on the following phases: i) extracting the question, ii) formulating an answer, and iii) checking/re-reading the message and sending it. All coaches will follow an intervention protocol that includes all guidelines for the provision of support.

Support will be provided by master level students in clinical psychology or individuals with a degree in the field of psychology. All coaches will undergo an intensive two-day training from an external company (‘Stichting E-hulp’), specifically focused on the delivery and methodology of online support. Coaches will be taught the basic principles for delivering online support and they will practice with case materials throughout the training. Monthly face-to-face supervision sessions will be organized by the main researcher, a psychologist and an experienced psychotherapist as a matter of routine professional and ethical care, as well as to reinforce adherence to the protocol. Individual supervision will also be provided to all coaches during their first month as online coach. Hereafter, the support sessions between coaches and participants will be regularly checked at random. A forum, in the form of a secured Facebook community, will be available for questions and discussion of scenarios in between the face-to-face supervision sessions, and coaches will furthermore be free to contact the supervisors at any time.

### Ethical precautions and crisis management

Coaches are instructed to refer participants who report suicidal ideation to the website 113Online (http://www.113online.nl); this organization aims to prevent suicide. It employs psychologists, psychiatrists, and a large group of fully trained volunteers, who are accessible via telephone and chat day and night. Furthermore, action is taken when screening or monitoring data show that a participant’s BMI is equal or lower than 15 or when a participant reports being engaged in self-induced vomiting, binge eating or use of laxatives at least once a day over the past four weeks. Subsequently, in case a participant is in condition one (basic Featback) or four (WLC, only screening data), the Featback team will send an e-mail with the message that his/her test scores indicate severe ED symptoms and that if he/she is not yet in treatment, we strongly recommend seeking professional help. In case a participant is in condition two or three, the Featback team will check whether one or more support sessions are planned for the week, and if not, will contact the participant with the message that we believe that his/her test scores indicate severe ED symptoms and that we strongly recommend to make use of one or more support sessions. In these support sessions, the alarm signals will function as a starting point for the conversation and participants will be stimulated to seek professional help. In case participants do not sign in for any support session during the week, the Featback team will send an e-mail as described for participants in condition one.

#### Assessments

All assessments are self-reported and will be conducted online. Table 1 depicts an overview of the assessment instruments that will be used throughout each stage of the study. Primary outcome measures are disordered eating behaviors and attitudes (SEED, EDE-Q) [23,27]. Secondary outcome measures contain ED-related quality of life (ED-QOL) [28], self-stigma of seeking help [29], self-esteem, mastery and support [30], symptoms of depression and anxiety (PHQ-4) [31], repetitive negative thinking (PTQ) [32], motivation to change [33,34], and user satisfaction, as well as compliance and help-seeking attitudes and behaviors. Cost-effectiveness will be evaluated by means of the reported quality of life (EQ-5D) [35] and medical and societal costs (Trimbos/iMTA Questionnaire for Costs Associated with Psychiatric Illness: TiC-P) [36].

**Table 1:** Overview of the assessment instruments used throughout each stage of the study

**Assessment**	**Screening**	**T0: Baseline**	**Weekly monitoring: even weeks**	**Weekly monitoring: uneven weeks**	**T1: Post-intervention**	**T2: 3-month follow-up**	**T3: 6-month follow-up**^2^
Weight Concern Scale (WCS)	X	-	-	-	-	-	-
Short Evaluation of Eating Disorders (SEED)	X	X	-	-	X	X	X
Short Evaluation of Eating Disorders II (SEED-II)	-	-	X	X	-	-	-
Demographics and other information	-	X	-	-	-	-	-
Monitoring questionnaire	-	-	X	X	-	-	-
Patient Health Questionnaire 4 (PHQ-4)	-	X	-	X	X	X	X
Perseverative Thinking Questionnaire (PTQ)	-	X	-	X	X	X	X
Eating Disorder Examination Questionnaire (EDE-Q)	-	X	-	-	X	X	X
Self-stigma of seeking help	-	X	-	-	X	X	X
Self-esteem, mastery and support	-	X	-	-	X	X	X
Eating Disorder-related Quality of Life (ED-QOL)		X	-	-	X	X	X
Motivation to change	-	X	-	-	X	X	X
Session Rating Scale (SRS)	-	-	X^1^	-	-	-	-
User satisfaction questionnaire	-	-	-	-	X^2^	-	-
Attrition follow-up question	-	-	X^3^	X^3^	X^3^	X^3^	X^3^
Help-seeking attitudes and behavior questionnaire	-	-	-	-	X	X	X
Quality of life (EuroQol: EQ-5D)	-	X	-	-	X	X	X
Trimbos/iMTA questionnaire for costs associated with psychiatric illness	-	X	-	-	X	X	X

^1^Only asked after participants’ first week of participation.

^2^Not sent to participants who are randomized to the waiting list control condition.

^3^Sent to participants who fail to complete the monitoring assessment, or the T1, T2 or T3 assessment, respectively.

The following variables will be tested as potential predictors or moderators of intervention response: demographic variables (age, gender, educational level), motivation to change (importance, ability and readiness to change), severity of ED symptoms (SEED, EDE-Q) [23,37], severity of symptoms of depression and anxiety (PHQ-4) [31], early working alliance (SRS) [38], and compliance.

Mediator variables and their corresponding dependent variable will be measured frequently throughout this study, at T0 and T1, as well as once every two weeks in between T0 and T1. The variables repetitive negative thinking (PTQ) [32] and symptoms of depression and anxiety (PHQ-4) [31] will be tested as mediators of intervention outcome, being symptoms of ED as measured by the SEED [23].

### Weight Concern Scale (WCS)

The WCS [39] assesses fear of weight gain, worry about weight and body shape, importance of weight, diet history, and perceived fatness. The WCS has demonstrated test-retest reliability and predictive validity [39] and was furthermore found valid in identifying students at risk for the development of an ED [40].

### Demographics and other information

A self-designed questionnaire will assess gender, age, educational level, country of origin and work situation, perceived severity levels of eating problems (including a question asking participants whether they have ever been diagnosed with an ED), and treatment status. Furthermore, the questionnaire asks participants how many days they have been sick during the previous three months, their average Internet usage during a typical week/day, and their average school/work performance during the previous three months.

### Eating Disorder Examination Questionnaire (EDE-Q)

The EDE-Q [27] has been developed as a self-report questionnaire version of the Eating Disorder Examination (EDE) [41], a semi-structured interview measuring ED psychopathology. The EDE-Q assesses both the frequency of core ED behaviors and the core attitudinal features of ED pathology over the past 28 days. Items assessing the latter are answered on a 7-point Likert scale ranging from 0 ‘not one day/not at all’ to 6 ‘every day/markedly’, and include questions about restraint, concerns about weight, concerns about shape and concerns about eating. A global score of eating psychopathology will be calculated by summing and averaging all the individual items. Higher scores are indicative of a higher ED psychopathology. The EDE-Q has demonstrated reliability and validity (see Berg et al., [42], for a review).

### Short Evaluation of Eating Disorders (SEED)

The SEED [23] is a brief self-report measure for the assessment of key ED symptoms. It assesses the three main symptoms of anorexia nervosa (degree of underweight, fear of weight gain, and distortion of body perception) and bulimia nervosa (amount of binge eating, amount of compensatory behavior, and over concern with body shape and weight). Total severity indexes can be calculated for each of the two diagnoses (range 0–3), with higher scores reflecting higher severity indexes. The behavioral measures (bingeing, excessive exercising, and compensatory behaviors) are assessed over the previous four weeks. The SEED has demonstrated construct validity and criterion-related validity, and was furthermore shown to be sensitive to symptom change [23].

Given that the SEED will be administered every week during the intervention period as well, the four-week timeframe for the behavioral measures is adapted to a timeframe of one week. This adapted questionnaire will be referred to as SEED-II.

### The Patient Health Questionnaire (PHQ-4)

The PHQ–4 [31] will be used to assess symptoms of depression and anxiety. The PHQ-4 consists of four items: two core anxiety items and two core depression items. Items can be answered on a 4-point Likert scale ranging from 0 ‘not at all’ to 3 ‘nearly every day’. A total score (range 0–12) can be calculated by summing the scores of all four items. Higher scores are indicative of a higher pathology. Factorial and construct validity were demonstrated for the PHQ-4 [31].

### Perseverative Thinking Questionnaire (PTQ)

The PTQ [32] will be used as a global measure of repetitive negative thinking (e.g., worry and rumination). The questionnaire consists of 15 items assessing the repetitiveness, intrusiveness, difficulties to disengage, and unproductiveness of repetitive negative thinking, as well as the degree to which it captures mental capacity. The scale of the items ranges from 0 ‘never’ to 4 ‘almost always’ and assesses how often each of the characteristics as described above applies to the participants’ thinking process. The Dutch PTQ demonstrated good internal consistency and satisfactory stability [32]. For the current study, we adapted the timeframe from ‘in general’ to ‘in the previous four weeks’ (T0, T1, T2, and T3 assessment) or ‘in the previous week’ (during the intervention period) respectively, in order to increase the ability to detect weekly or monthly change.

### Self-stigma of seeking help

The 10-item self-stigma of seeking help [29] questionnaire will be used to assess one’s self-stigma towards seeking psychological help. The questionnaire was developed to measure concerns about the loss of self-esteem and an overall sense of loss of value a person would feel if he/she would decide to seek help from a psychologist or any other mental health professional. Answers can be rated on a 5-point Likert scale ranging from ‘strongly disagree’ to ‘strongly agree’. Higher scores reflect higher self-stigma or a more negative stigma toward seeking psychological help. The questionnaire was found to have good psychometric properties [29].

### Self-esteem, mastery and support

Starting from existing instruments, Bovier et al. [30] used factor analyses to develop four brief scales for the measurement of self-esteem (four items), mastery (four items), affective social support (two items), and confident/problem solving social support (four items). Affective support refers to the availability of people who express emotional involvement with, and care for a person, whereas confident/problem solving support refers to the availability of an individual one can confide in and receive advice from when a challenging situation occurs [30]. Items of all four brief scales can be answered on a 5-point Likert scale. Higher scores represent higher self-esteem, mastery, affective- and confident support, respectively. All four scales were found to demonstrate good internal and construct validity [30].

### Eating Disorders Quality of Life (ED-QOL)

The ED-QOL [28] is a disease-specific health-related quality of life measurement designed for individuals with ED symptoms. The ED-QOL consists of 25 items, assessing the influence of eating behaviors/body weight in four subscales: psychological (nine items), physical/cognitive (six items), financial (five items), and work/school (five items). A total score can be calculated by averaging the items of the four subscales. Higher scores are indicative of a lower quality of life. The ED-QOL demonstrated good convergent and discriminative validity, as well as test-retest reliability [28].

### Motivation to change

Three items will be used to assess participants’ motivation to change [33,34]: their perceived importance to change*,* their perceived ability/confidence to change, and their readiness to change. Questions can be answered on a 10-point Likert scale.

### Session rating scale (SRS)

The SRS [38] will be used to measure the working alliance between participants and their coaches, as well as to measure the perceived degree of support from the automated feedback messages and coaches, respectively. The SRS consists of four items that assess four aspects of the working alliance: the relational bond, the degree to which desired goals and topics of the individual are discussed, an evaluation of the therapist’s approach or method used, and an evaluation of the overall perception of the session by the individual. Instead of using a visual analogue scale, an 11-point Likert scale will be used to answer each of the four items, with ‘0’ depicting a negative response and ‘10’ depicting a positive response. The SRS demonstrated a high test-retest and internal consistency reliability, as well as an acceptable validity [38].

### User satisfaction questionnaire

A self-designed questionnaire was developed to assess the user satisfaction of Featback, such as the perceived quality of the support, whether Featback helped them to deal more effectively with their eating problems, and how satisfied they are with Featback in general. Participants are also asked to rate the individual components of Featback and to provide negative and positive comments about Featback.

### Compliance

Two measures of compliance will be extracted from the database. The first measure of compliance will be the number of times a participant has completed the weekly monitoring assessment. The second measure of compliance will be the number of times a participant has completed a monitoring assessment after a completed assessment in the previous week. This second measure of compliance is important because Featback is only programmed to compare obtained results with those of the previous week, not with those completed at earlier points in time. Thus, in case one has missed a monitoring assessment, but completes the next monitoring assessment a week later, results of the completed assessment cannot be compared to the previous results (e.g., results of the previous week are missing). Subsequently, progress or deterioration cannot be accurately monitored, which in turn can produce more general and less individually tailored feedback. To be able to further investigate dose–response relationships, the number of support sessions a participant has received will be recorded as well.

### Attrition follow-up questions

According to Eysenbach [43], there are two different processes of attrition; “dropout attrition” refers to participants being lost to follow‒up, thus not returning follow‒up questionnaires and “non-usage attrition” refers to participants who stop using the intervention. To be able to investigate the reasons for both dropout and non-usage attrition, two attrition follow-up questions are designed: one in case participants fail to complete monitoring assessments and another one in case participants fail to complete the T1, T2, or T3 assessment.

### Help-seeking attitudes and behavior questionnaire

Several questions will be used to assess participants’ help-seeking attitudes and behavior, for example, whether participants generally believe that professional help is beneficial and whether they believe that they need to seek professional help themselves. Furthermore, the questionnaire investigates whether participants intended to seek professional help and whether they actually sought professional help. Regarding the latter, the participants are also asked whether Featback has contributed to the decision to seek help.

### Quality of life

The EuroQol (EQ-5D) [35] generic health index is a standardized, patient-completed instrument which consists of five dimensions: mobility, self-care, usual activities, pain/discomfort, and anxiety/depression. Each dimension can be rated on three levels (no problems, some problems, and extreme problems). Thus, 243 distinct health states are defined, each with a unique utility score, ranging from 1 (‘perfect health’) to 0 (‘death’).

### Direct medical costs

For calculating the total direct medical costs, the Trimbos/iMTA questionnaire for Costs associated with Psychiatric Illness (TiC-P) [36] will be used. The TiC-P measures the utilization of medical treatment such as the number of contacts with the general practitioner and multiple other care providers (e.g., medical specialists and paramedics) during the last three months, as well as the medication used. The costs will be calculated using the Dutch guidelines for cost calculations in health care [44]. Reference unit prices of the corresponding health care services will be applied. The cost-utility will be calculated by relating the difference in direct medical costs per patient receiving Featback and care as usual (WLC) to the difference in terms of quality adjusted life years gained (cost-utility), yielding a quality adjusted life years estimate.

### Sample size

The sample size calculation is based on an expected small between-group effect size (Cohen’s *d* = 0.32) [45]. The calculation is conducted by the software program Power Analysis and Sample Size (PASS, 2008). The primary analysis will concern the hypothesis that the average post-intervention level of eating pathology in the waiting list control condition is higher than the average level of eating pathology in the three active intervention conditions. Assuming an alpha of 0.05 and a power of 0.80 (β-1) in a one-way ANOVA study, we need sample sizes of 79 participants in each of the four groups whose means are to be compared using a planned comparison. Taking into account a baseline variable (e.g., T0 assessment) for which we assume a Pearson correlation of 0.5 with the outcome variable, and thus explaining 25% of the variance of the outcome variable, the sample size per group can be reduced by 25% and is thus calculated as 0.75 × 79 = 60 per group. In order to account for dropout, recently reviewed to be approximately 30% for Internet-based interventions [46], the definitive number of participants we will need to recruit is 10/7 × 60 = 86 participants per group, resulting in 344 in total.

### Statistical analyses

All statistical analyses will be performed in SPSS version 19. A two-tailed significance level of *α* = 0.05 will be used throughout the analyses. Both intent-to-treat and completers analyses will be conducted. Intent-to-treat analyses will include every participant who is randomly allocated to the intervention, regardless of withdrawal or deviation from the protocol [47]. Someone will be considered a completer in case he/she has completed both T0 and T1 assessments, and at least five monitoring assessments.

Pre-treatment differences between the conditions will be investigated using *χ*^*2*^ tests for categorical variables and ANOVAs for continuous variables. Linear mixed model analyses will be used to investigate the effectiveness and maintenance effects of Featback. Time contrasts will be created by means of dummy-coding. Within- and between-group effect sizes (Cohen’s *d*) [48] will be calculated based on the pooled standard deviation. As recommended by Frazier, Tix and Barron [49], potential categorical predictor or moderator variables will be dummy coded, and potential continuous predictor or moderator variables will be standardized. A significant two-way interaction between predictor and time indicates a predictor effect. A significant three-way interaction between time, condition and moderator, indicates a moderator effect. Significant moderator effects of continuous variables will be interpreted by dichotomizing the moderator variable into subgroups of participants who score either low or high (e.g., below or above the sample mean) on the moderator variable. Separate mixed model analyses will then be repeated to examine interactions between condition and time within the low and high subgroups.

A cross-lagged panel design will be used to determine whether changes in mediator variables predict changes in ED symptoms, and not vice versa, as described by Burns et al. [50]. For all outcome and mediator variables, residualized change scores will be calculated for baseline (T0) to mid-intervention (week 4), as well as for mid- (week 4) to post-intervention (T1: week 8). Hereafter, hierarchical regressions will be performed, with mid- to post-intervention standardized change of the primary outcome measure as dependent variable, pre- to mid-intervention standardized change of the primary outcome measure, and mid- to post-intervention standardized change of the mediator variable as independent variables in the first step. In the second step, pre- to mid-intervention standardized change of the mediator variable will be entered into the regression equation. In addition, the inverse association (whether changes in the primary outcome variable predict changes in the mediator variable) will be tested and should not be significant.

### Cost utility analysis

The aim of this economic evaluation is to assess the cost utility of Featback compared to the WLC condition. For examining the cost-effectiveness of Featback, the direct costs and quality of life scores will be calculated using SPSS, and normalized using Box-Cox transformations and power transformations. In case of missing data, the missing values in direct costs and quality of life scores per time unit will be imputed with a Markov Chain Monte Carlo Multiple Imputation in SAS. Different variables, like scores on the WCS and SEED, age and gender will be included to get a better estimate. Propensity scores may be used to correct for baseline differences between groups. The uncertainty in the analysis will be assessed using bootstrapping in Excel. The results of the economic evaluation will be expressed in a cost-effectiveness acceptability curve. The acceptability curve illustrates the probability that the cost-effectiveness ratio will be accepted for different cost limits.

## Discussion

One of the strengths of this study is the evaluation of the (cost-) effectiveness of the intervention, as well as the evaluation of the (cost-) effectiveness of the different levels of support. To our knowledge, this has not yet been investigated in the field of ED.

Both a strength and a limitation of this study is that participants only have to meet three eligibility criteria (16 years of age or older, mild ED symptoms or at risk for the development of an ED, and internet access). A possible limitation could be the influence of comorbid disorders or the use of co-interventions or medication on study outcome measures; a possible strength is that many individuals who suffer from an ED have comorbid mental health problems such as depression, anxiety, substance dependence, and personality disorders [51,52]. Therefore, the broad inclusion criteria may well bear a close resemblance to reality, enhancing the external validity of the results, as well as being consistent with the aim of an applicable and easily accessible intervention for a broad population of individuals with symptoms of ED.

Another characteristic of this study that is both a strength and a limitation, is that measurements are conducted solely online. The advantages that come with online assessments are a reduction in research costs, maximization of the accessibility of participation, and participant anonymity. However, the lack of face-to-face assessment(s) also means a lack of a diagnostic interview, and may furthermore reduce the commitment to the study and the intervention. In order to maximize compliance, motivational reminders will be sent repeatedly, and individuals who complete all study assessments will take part in a lottery including gift vouchers and an iPod.

One of the limitations of this study concerns the lack of a 6-month follow-up for participants in the WLC, given that they will receive Featback with infrequent support from a coach after T2. This means that the relative long-term effectiveness of Featback, as compared to a WLC condition, cannot be examined. Fortunately, the longer-term follow-up data of the different forms of feedback (without support, with infrequent support, and with frequent support) will be available and examined.

Another limitation of this study is that due to the research questions and corresponding design of this study (a randomized controlled trial), it is impossible to fully preserve the stepped-care nature of Featback. Participants will be randomized to Featback without support or to Featback with infrequent or frequent support, which might not (always) match the preferences of participants.

## Trial status

Patient enrollment was completed on the 17th of June, 2013.

## Abbreviations

ED: Eating disorders; EDE-Q: Eating disorder examination questionnaire; ED-QOL: Eating disorder-related quality of life; PHQ-4: Patient health questionnaire; PTQ: Perseverative thinking questionnaire; SEED: Short evaluation of eating disorders; SRS: Session rating scale; TiC-P: Trimbos/iMTA questionnaire for costs associated with psychiatric illness; WLC: Waiting list control; WSC: Weight concern scale.

## Competing interests

The authors declare that they have no competing interests.

## Authors’ contributions

Authors JA, AD, PS and EF contributed to the design of this study and helped to draft the manuscript. LH drafted the health economics evaluation/cost-utility part of this study protocol. All authors read and approved the final manuscript.
